# Physicians’ Protective Association

**Published:** 1886-05

**Authors:** 


### EDITORIAL.

PHYSICIANS' PROTECTIVE ASSOCIATION.

Attention was called to the importance of establishing a PHYSICIANS' PROTECTIVE ASSOCIATION in an editorial appearing in the January number, 1885, of this JOURNAL. The note was suggested by an eminent surgeon, whose knowledge of the conditions of professional life in Chicago is adequate, and is worthy of reproduction at this time:

The object of the association is to protect physicians from groundless charges of malpractice. It is proposed that all physicians, interested in the subject, unite in the establishment of a fund for the defense, in cases of malpractice, and the election of a board of trustees to investigate individual cases, decide on their merits, and secure counsel. This matter is one of interest to every active medical practitioner, and no apology is demanded for calling the attention of the profession to the scheme before it has assumed definite proportions.

The advantages of such an association are obvious. The effect upon the community would be of the most salutary character. We have in Chicago all the conditions for the production of malpractice suits. It is scarcely necessary to direct attention to our large pauper population, native and foreign, the frequent occurrence of all forms of accidents,—in connection with the fabulous number of railroads that center in the city, and the numerous manufacturing industries,—the crowded state of the legal profession. Within the past twelve months, ten or twelve suits for alleged malpractice have been instituted against as many reputable physicians by pauper patients, backed by irresponsible legal advisers. Not one charge was sustained. The physician, however, has in each case lost time and money, while his professional reputation has suffered from newspaper notoriety. It is an alarming fact that there is not a physician in Chicago of such eminence as to preclude the possibility of indictment for malpractice, at the instigation of a dispensary patient! An organization of the nature indicated would render irresponsible representatives of the legal profession less eager to share the "spoils" with pauper patients.

Such an association would have a favorable influence upon the profession. Medical gentlemen would be more charitable and truthful in their criticisms of the treatment of cases in regard to which they possessed no accurate information.

Many of the recent malpractice suits in Chicago have been instigated by the careless word of a brother practitioner, who had absolutely no knowledge as to the conditions of the case. When moral considerations do not restrain one practitioner from criticizing the treatment of a brother, the laws of the land render it criminal for him to utter a falsehood.

At a meeting of the CHICAGO MEDICAL SOCIETY, DR. EDMUND J. DOERING read an able paper upon the same subject, which elicited a spirited discussion. The weight of professional opinion, however, was in favor of a PROTECTIVE ASSOCIATION. Subsequently, timely and favorable editorials upon this topic have appeared in the *Journal of the American Medical Association*, January 30th, 1886, and the *Medical Record*, February 27th, 1886.

Now that the merits and demerits of the proposed association have been thoroughly discussed, it is time that a meeting of those interested in the movement shall be called.

